# Concanavalin a Grafted Nanoemulsions for Nasal Delivery: Preliminary Studies with Fluorescently Labelled Formulations

**DOI:** 10.3390/ma17204959

**Published:** 2024-10-11

**Authors:** Merve Mışraklı, Sebastiano Antonio Rizzo, Valentina Bordano, Annalisa Bozza, Luca Ferraris, Elisabetta Marini, Elisabetta Muntoni, Maria Teresa Capucchio, Anna Scomparin, Luigi Battaglia

**Affiliations:** 1 Faculty of Pharmacy, Ege University, Erzene Street, Ankara Avenue, No 172/98, 35040 Izmir, Türkiye; misrakli20@gmail.com; 2 Department of Drug Science and Technology, University of Turin, Via Pietro Giuria 9, 10125 Turin, Italy; sebastianoantonio.rizzo@unito.it (S.A.R.); valentina.bordano@unito.it (V.B.); annalisa.bozza@unito.it (A.B.); luca.ferraris@unito.it (L.F.); elisabetta.marini@unito.it (E.M.); elisabetta.muntoni@unito.it (E.M.); 3 Department of Veterinary Sciences, University of Turin, Largo Paolo Braccini, 2, 10095 Grugliasco, Italy; mariateresa.capucchio@unito.it

**Keywords:** nanoemulsion, nasal, permeation enhancer, lectin

## Abstract

Nasal delivery is a non-invasive strategy for effective drug delivery. Nevertheless, in order to promote drug uptake by the nasal mucosa, it is fundamental to increase its residence time in the administration site. To this aim, nano-sized drug delivery systems are widely exploited. Within this context, the commercially available nanoemulsion for parenteral nutrition is a biocompatible, safe and clinically approved vehicle for drug delivery. Furthermore, the nanodroplet surface can be modified via a well-established protocol to graft Concavalin A, a lectin capable of improving the mucosal adhesion, by binding to the α-mannose and α-glucose residues of the mucosal glycocalyx. The obtained targeted formulation is able to induce haemagglutination, as opposite to non-modified nanoemulsion. Furthermore, the ConA grafting maintains the physicochemical properties of the nanodroplets (size~230 nm, Z < −35 mV) and does not interfere with the loading of the Rose Bengal fluorescent probe. Fluorescently labelled ConA grafted nanodroplets showed enhanced permeation and accumulation in ex vivo bovine nasal mucosa. This study is a proof of concept that Concanavalin A can be used to decorate the surface of nanodroplets, acting as a permeation promoter.

## 1. Introduction

As an alternative administration route, intranasal drug delivery shows several advantages over conventional ones: fast onset of action, potential nose-to-brain targeting and ease of administration [1,2]. Nonetheless, the major limitations for nasal drug delivery are: the overcoming of the so called “nasal valve” and the mucociliary clearance at the nasal mucosa. The former is an anatomical constriction located between the caudal and the upper cartilage and the *septum*, which prevents the air flow entrance in the bone nasal cavity via the vertical pyriform aperture [3]. The mucociliary clearance, instead, reduces the residence time of drugs at the nasal mucosa: with the cilia beating at a frequency of 1000 beats per minute, mucus moves from the anterior to the posterior part of the nasal cavity at a rate of 5 mm per minute.

Therefore, suitable strategies should be adopted to meet the above-mentioned concerns. While “nasal valve” overcoming can be optimized by the employment of suitable devices/inhalers, the improvement of the residence time at the nasal mucosa can be achieved by bio-adhesive nanocarriers with optimized composition [4,5,6]. Of note, an innumerable series of nanocarriers have been engineered over the last decades, including hyper-crosslinked and hollow polymeric nanostructures, endowed to multiple drug loading and release capabilities, and several potential surface functionalization strategies are available [7,8]. However, the clinical translation of the most recent and innovative nanoparticles is frequently slowed by scale-up and regulatory concerns.

Within this context, injectable nanoemulsions for total parenteral nutrition, endowed with a safe history of clinical usage, have been recently re-proposed as nasal nanocarriers [9]. Indeed, they are composed by a biocompatible oily core stabilized by surfactants: the lipid component favours the loaded drug partition in the nasal mucosa, while the surfactant enhances the penetration of biological membranes [10]. Specific sprays/inhalers have been engineered and are available on the market to deliver liquid nasal formulations beyond the nasal valve [11,12,13]. In particular, due to their reduced size and low viscosity, nasal nanoemulsion can be effectively delivered by means of multi-dose pumps, without any further modification [14]. Nonetheless, quick mucosal uptake of intact nanodroplets is likely hampered by their relatively large size [15].

Hence, surface functionalization of the lipid nanodroplet with chemical moieties capable of establishing specific bonds with the surface of the nasal mucosa is worthy of investigation. To this aim, in this experimental work Concanavalin A (ConA), a lectin originally extracted from the jack-bean, was selected as the ligand to be grafted onto the nanodroplet surface because it efficiently binds to the α-mannose and α-glucose residues of the mucosal glycocalyx [16]. By this way, ConA should increase the intimate bio-adhesion of the nanodroplet, thus improving the release of its cargo at the mucosal site. The lipid matrix of functionalized nanoemulsions was labelled with the fluorescent probe Rose Bengal. Then, formulations underwent physico–chemical characterization and ex vivo studies on isolated bovine nasal mucosa, in order to assess the permeation and accumulation of the fluorescent probe, taken as a model of potential drug candidates.

## 2. Materials and Methods

### 2.1. Chemicals

Sodium citrate, citric acid, disodium hydrogen phosphate, sodium dihydrogen phosphate, hydrochloric acid, potassium chloride and sodium chloride were purchased from Carlo Erba (Cornaredo, Italy). Concanavalin A (ConA) freeze-dried, prepared by chromatography on Sephadex^®^, carbohydrate < 0.1% was purchased from Pharmacia Fine Chemicals (AB Uppsala, Sweden). Sephadex^TM^ G-25 medium was purchased from GE Healthcare (Chicago, IL, USA). 98% 2-iminothiolane, anhydrous > 99% dimethyl sulfoxide (DMSO), HPLC grade methanol and 98% tris(2-carboxyethyl) phosphine (TCEP) were purchased from Sigma-Aldrich (St. Louis, MO, USA). HPLC grade acetonitrile (ACN), 99% glucose, HPLC grade isopropanol (IPA) and 99% trifluroacetic acid (TFA) were purchased from Alfa Aesar (Haverhill, MA, USA). 20% Lipofundin^®^ MCT (IL) was purchased from B. Braun (Melsungen, Germany). 97% Cystein-DL was purchased from D-7924 (Steinheim, Germany). Rose Bengal was purchased from BDH (Dubai, UAE). Milli-Q water (for deionized water) was purchased from Merck (Darmstadt, Germany). Pullulan (15.0 to 180.0 mPas 10%, H_2_O, 30 °C) was purchased from TCI Europe (Zwijndrecht, Belgium).

### 2.2. Cells

Rat red blood cells (RBC) were collected from euthanized animals in heparinized tubes (owing to protocol number 56105.N.WSP, approved by the Italian Ministry of Health on 14 July 2023) and diluted up to 2% in phosphate-buffered saline (PBS): 200 μL of rat blood in 10 mL PBS.

### 2.3. Methods

#### 2.3.1. Linkers Synthesis and Characterization

N-Octadecil-3-Maleimido-Benzamide (ST-MBS) was synthesized and characterized according to a previously established method [17,18].

#### 2.3.2. IL Functionalization with ConA

ConA thiolation was preliminarily performed. To this aim, three stock solutions were prepared separately: (1) 2 mg ConA in 1.6 mL 0.1 M phosphate buffer pH = 7.4; (2) 6.4 mg TCEP (a mild reducing agent used to prevent thiol groups dimerization to disulfide bonds) in 1 mL distilled water; (3) 1.6 mg 2-iminothiolane in 1 mL distilled water. The thiolation was carried out as follows: stock solution 1 was mixed with 200 μL of stock solution 2 and 200 μL of stock solution 3 and kept for 1 h under magnetic stirring. Meanwhile, Sephadex G-25 column for gel centrifugation was prepared as follows: 7 g Sephadex^TM^ G-25 medium was suspended in 42 mL distilled water, gently mixed and hydrated in the oven at 90 °C for 1 h, and then used to pack a 10 mL syringe column, which was centrifuged at 1500 rpm until dryness was achieved. The aforementioned thiolation reaction mixture was then gel centrifuged through the Sephadex G-25 column [16,17,18].

The reaction between thiolated ConA and ST-MBS loaded IL was performed as follows: 0.4 mg ST-MBS was dissolved in 20 μL DMSO, and this solution was added to 2 mL IL under vortexing. Gel centrifuged thiolated ConA (2 mL) was added to ST-MBS loaded IL (1 mL) and kept reacting overnight. Then, 1.6 mg cysteine was dissolved in 1 mL distilled water. 200 μL of such solution (corresponding to 0.32 mg cysteine) was added to the reaction mixture, in order to saturate excess maleimide, and left under magnetic stirring for 4 h. Then, ConA grafted IL was isolated from the reaction mixture by ultracentrifugation for 5 min in 4 microcentrifuge tubes (1 mL) at 26000 rpm (64R Allegra Centrifuge, Beckman Coulter, Brea, CA, USA) and resuspension in 2 mL distilled water after a short sonication (10 s) [17,18]. The supernatant from the reaction mixture was also kept separately for further analysis.

#### 2.3.3. Particle Size, Shape, Zeta Potential and ConA Content of Nanoemulsions

The dynamic light scattering technique (DLS; 90 Plus, Brookhaven, NY, USA) was used to determine the mean droplet size, polydispersity index (PDI) and Zeta potential of the IL-based formulations, at 25 °C and in triplicate. Measurement angles were 90° for particle size and 15° for Zeta potential. Optical microscopy was performed by a DM2500 microscope (Leica Microsystems, Wetzlar, Germany) equipped with a Moticam 480 camera (Motic, Barcelona, Spain).

ConA grafting was quantified by means of reverse-phase high-pressure liquid chromatography (RP-HPLC) [12]. Prior to HPLC injection, ConA was extracted as follows: 50 μL of ConA grafted IL was diluted with 100 μL 70% IPA, 20% ACN, 10% water, 0.1% TFA and centrifuged at 26,000 rpm for 15 min. The same procedure was applied to the supernatant of the reaction mixture to quantify unreacted thiolated ConA.

#### 2.3.4. Haemagglutination Test

The activity of ConA, either as free or IL grafted, was checked by means of a haemagglutination test with 2% rat RBC [16]. In the former case, a ConA stock solution was prepared by dissolving 1 mg ConA in 1 mL PBS. Such solution was mixed with 1 mL of 2% rat RBC in four different proportions: (1) 70 μL (corresponding to 70 μg ConA); (2) 130 μL (130 μg ConA); (3) 260 μL (260 μg ConA); (4) 540 μL (540 μg ConA). The solutions were kept for 30 min in incubation at 37 °C. Then, haemagglutination was visually evaluated by RBC sedimentation and optical microscopy, and it was quantified with a spectrophotometer (Lambda 2 UV–vis spectrophotometer, Perkin-Elmer, Waltham, MA, USA) at λ = 700 nm after dilution of the samples to 3 mL with PBS. Untreated 2% RBC were used as the positive control. The % haemagglutination was calculated as follows:

$$\%\;\mathrm{haemoagglutination}=100\;\times \left(1-\frac{{\mathrm{UA}}_{\mathrm{treated}\;\mathrm{RBC}}}{{\mathrm{UA}}_{\mathrm{control}\;\mathrm{RBC}}}\right)$$

In the case of IL grafted ConA, the ratio between 2% RBC and nanoemulsion was based upon the quantified surface grafted ConA: haemagglutination was evaluated by visual observation and optical microscopy.

#### 2.3.5. ConA-Mediated Coalescence Assay

Coalescence was assessed using a 2% starch mixture. Briefly, 0.5 mL of ConA grafted IL was added to 60 μL of 2% starch mixture gradually until reaching 500 μL. Droplet enlargement was evaluated by optical microscopy and DLS. Blank IL was used as the control.

#### 2.3.6. Nanoemulsions Fluorescence Labelling with Rose Bengal

A 5 mg/mL stock solution of the fluorescent probe Rose Bengal in DMSO was prepared. Such solution was added either to blank IL or ConA grafted IL with the ratio of 20 μL stock solution to 1 mL nanoemulsion (final Rose Bengal concentration in the nanoemulsion 100 μg/mL). Labelled nanoemulsions were characterised with optical microscopy, DLS and Zeta potential.

Rose Bengal percent entrapment efficiency (EE%), expressed as the ratio between the probe loaded in the oil droplets *vs* the total present in the nanoemulsion, was evaluated as follows. A total of 500 μL Rose Bengal-labelled nanoemulsion was centrifuged at 26,000 rpm for 5 min: the floating pellet (constituted by the oil droplets) was separated from the underlying supernatant (that is the water outer phase). The pellet was resuspended with 500 μL of distilled water. Then, both resuspended pellet and supernatant were diluted with methanol until 5 mL in order to extract the fluorescent probe. Such mixtures were centrifuged at 26,000 rpm for 5 min, and the clear supernatant was analysed with a spectrophotometer at λ = 550 nm. The entrapment efficiency was calculated as follows, based on the recorded units of absorbance (UA):

$$\mathrm{EE}\%=\frac{{\mathrm{UA}}_{\mathrm{pellet}}}{{\mathrm{UA}}_{\mathrm{pellet}}+{\mathrm{UA}}_{\mathrm{supernatant}}}\times 100$$

#### 2.3.7. Permeation through Nasal Mucosa

The permeation and accumulation in the nasal mucosa were studied by using a multi-compartment rotating cell. Such a device is constituted by two matching Plexiglass plates: in each of them several 2 mL chambers are embedded (Figure S1). Thus, each of the two facing chambers in the plate, separated by the nasal mucosa, serve as the donor and the receiving phase, respectively.

Bovine nasal mucosa was obtained from animals regularly slaughtered at the teaching slaughterhouse of the Department of Veterinary Sciences, University of Turin, within two hours from death. Animal nasal mucosa (except the septum part) was excised and separated from the superior nasal concha by animal pathologists. Tissues were stored in PBS, until further analysis.

Then, 1 mL of Rose Bengal-labelled formulation was diluted with 1 mL water and put in contact with the mucosa. Two separate sets of experiments were performed: in the first one, the receiving phase was constituted by 2 mL of PBS, and in the second one, by 2 mL of 1% pullulan (used as a chemo-attractant agent) in PBS. At scheduled times, 0.2 mL of the receiving phase was withdrawn and replaced with fresh PBS. At the end of the experiment, the mucosa was washed with tap water; Rose Bengal was extracted with 5 mL methanol. Quantification of the permeated probe was performed through spectrofluorimetry by using a Multilabel Plate Reader (Victor3 1420, Perkin Elmer, Waltham, MA, USA): λ_exc_ = 485 nm, λ_em_ = 540 nm. Quantification of the probe accumulated in the mucosa was performed by RP-HPLC.

### 2.4. Reverse Phase (RP) Cromatographic Analysis

#### 2.4.1. ConA RP-HPLC

RP-HPLC analysis for ConA protein was carried out with a Shimadzu LC20 HPLC, equipped with a LC-20 AB binary pump, an SPD-20AV UV–vis detector, a CTO-10AS oven, and Class LC-20 software v.1.25 SP5 (Shimadzu, Tokyo, Japan). Temperature was set at 75 °C, and a 300 Å pore C8 column (tracer excel 25 × 0.4 cm, Tecnokroma, Barcelona, Spain) was used. The gradient was performed between eluent A (0.1% TFA) and eluent B (70% IPA, 20% ACN, 10% Water, 0.1% TFA): 0 min: 90% A; 15 min: 40% A; 20 min: 40% A; 23 min: 90% A. The flow rate was maintained at 1 mL/min. The UV detector was set at 220 (for quantification) and 280 nm (for identification) [18,19,20]. A calibration curve was obtained at 220 nm between 1000 and 20 μg/mL (R^2^ = 0.9999, LOD = 1.6 μg/mL; LOQ 5.4 μg/mL).

#### 2.4.2. Rose Bengal RP-HPLC

HPLC analysis was performed with a YL9110 Quaternary Pump, equipped with a YL 9160 diode array detector (PDA-Yang Lin, Anyang, Republic of Korea) and a Shimadzu RF-20 fluorescence detector (Shimadzu, Tokyo, Japan), linked to Clarity software v.3.0.4.444 for data analysis (Yang Lin, Anyang, Republic of Korea). RP-HPLC analysis for Rose Bengal was carried out using a C18 column (Mediterranea Sea 25 × 0.4 cm, Tecnokroma, Barcelona, Spain). The gradient was performed between water (A) and methanol (B): 0 min: 95% A; 5 min: 50% A; 10 min: 50% A; 12 min: 95% A. The fluorimeter was set as follows: λ_exc_ = 485 nm, λ_em_ = 540 nm. The flow rate was maintained at 1 mL/min. The Rose Bengal retention time was 7.5 min. A calibration curve was obtained between 500 and 50 ng/mL (R^2^ = 0.9993, LOD = 5.1 ng/mL; LOQ 15.4 ng/mL).

### 2.5. Statistical Analysis

Data were expressed as mean ± S.E.M. Statistical analysis was performed according to the unpaired *t*-test with Prism GraphPad v5.0.

## 3. Results

### 3.1. Characterisation of Free ConA

**Figure 1:**
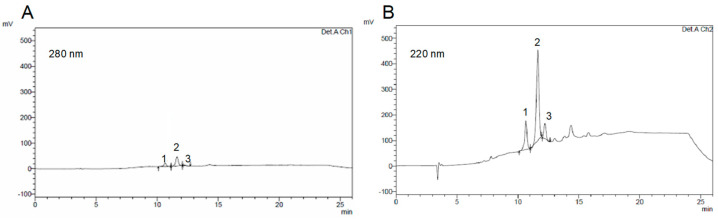
HPLC of free Concanavalin A (ConA): (**A**) 280 nm detection; (**B**) 220 nm detection. Peak retention times: peak 1: 10.6 min; peak 2: 11.6 min; peak 3: 12.2 min.

The chromatogram of free ConA (1 mg/mL dissolved in 0.01 M hydrochloric acid) is shown in Figure 1. Three peaks were detected, with one prominent compared to the others, in a similar way to RP-HPLC chromatograms of lectins, obtained with acid elution [21,22,23]. The attribution of such peaks to specific glycoprotein isoforms or multimers, constituting the ConA structure, goes beyond the scope of this work. In the literature it was hypothesized that lectins are composed of different types of subunits with similar MW, thus justifying the multiplicity of peaks eluted in RP-HPLC. Nonetheless, it is well known that ConA, and lectins in general, are composed by four subunits, and that the co-existence of tetrameric, dimeric, and monomeric forms is regulated by pH, with the low MW forms prevailing in acid conditions. Protein elution in RP-HPLC is regulated by the interaction of their hydrophobic foot with the 300 Å pores, present on the surface of the stationary phase granules [24]. Therefore, since the single subunits and their multimers should expose different hydrophobic foots, it might not be excluded that monomer, dimer and tetramer can be separated during this chromatographic elution.

**Figure 2:**
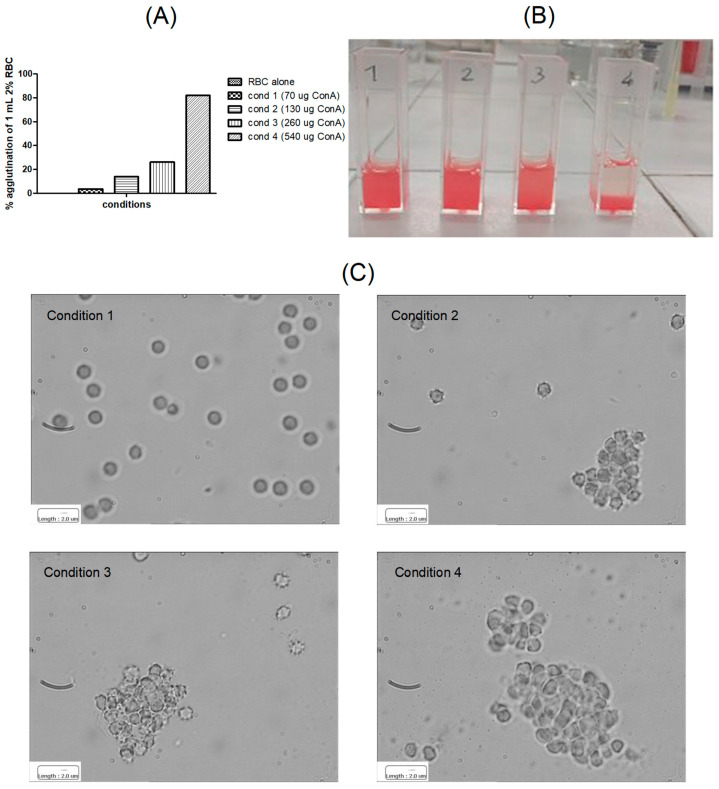
Agglutination assay for free Concanavalin A (ConA). (**A**) % agglutination in spectrophotometry; (**B**) visual observation; (**C**) optical microscopy. Reported scale bar: 2 μm.

In Figure 2 RBC agglutination in the presence of free ConA is shown. A suitable ratio between ConA and RBC is needed to obtain the agglutination. Therefore, 2% RBC were used, based upon literature evidence [16], despite RBC being typically employed at 4.45% for haematocrit. It can be noticed that in condition 4 (higher concentration of ConA) the agglutination is maximised.

### 3.2. Nanoemulsions Characterization

**Figure 3:**
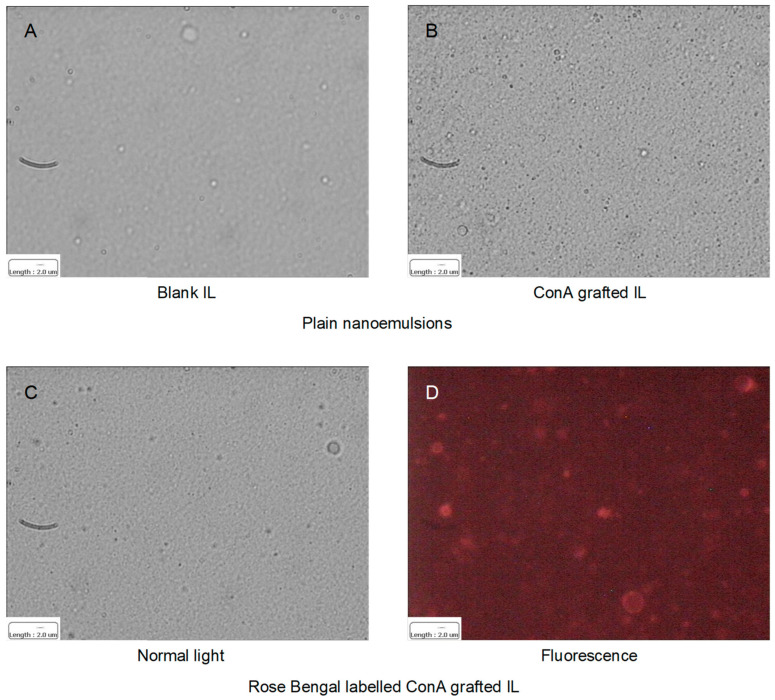
Optical microscopy of nanoemulsions. (**A**) Blank IL—normal light; (**B**) ConA grafted IL—normal light; (**C**) Rose Bengal labelled ConA grafted IL—normal light; (**D**) Rose Bengal labelled ConA grafted IL—fluorescence. Reported scale bar: 2 μm. The fluorescence image was captured with the rhodamine filter (λ_exc_ = 515; λ_em_ = 560, N2.1 filter cube, Leica Microsystems, Wetzlar, Germany). Abbreviations: ConA: Concanavalin A; IL: 20% Lipofundin^®^ MCT.

In Figure 3 and Table 1 the characterization of blank and ConA grafted IL, plain or fluorescently labelled with Rose Bengal, is shown.

No aggregates were shown in optical microscopy. Particle size was maintained both after ConA grafting and fluorescence labelling, with a minor influence on Zeta potential. Besides IL grafted ConA, less than 100% (43.9 ± 2.8%) unreacted thiolated ConA was recovered in the supernatant of the reaction mixture. This might mean that nearly half of the initial ConA was either retained/adsorbed in the gel centrifugation process, or undergoing aggregation in the supernatant of the reaction mixture, owing to disulfide bond formation: in this case the dimerised ConA would exceed the size range limit for RP-HPLC. However, this phenomenon would not affect the final reaction yield, since only a small amount ConA (64.6 ± 9.7 μg/mL) can be grafted on IL surface. Rose Bengal EE% was slightly increased by ConA grafting. Indeed, in the case of blank IL, no specific fluorescence was detected with optical microscopy in correspondence of oil droplets, likely due to both photo-bleaching and to a relevant partition of the fluorescent probe into the outer phase. On the contrary, when Rose Bengal was loaded in ConA grafted IL dotted fluorescence was detected in correspondence of oil droplets.

**Figure 4:**
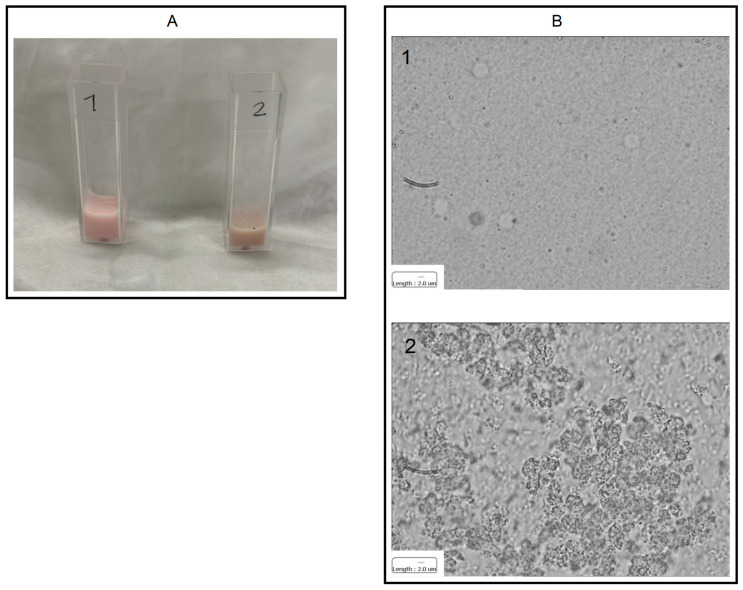
Agglutination assay for IL grafted ConA: (**A**) visual observation; (**B**) optical microscopy. Condition 1: IL + 2% RBC; condition 2: ConA grafted IL + 2% RBC. Reported scale bar: 2 μm. Abbreviations: ConA: Concanavalin A; IL: 20% Lipofundin^®^ MCT; RBC: red blood cells.

For the haemagglutination assay, the ratio between 2% RBC and ConA grafted IL was based upon ConA content in functionalized IL. Two conditions were used: (1) 0.5 mL ConA grafted IL (64.6 ± 9.7 μg/mL ConA) with 60 μL 2% RBC; (2) 0.5 mL ConA grafted IL (64.6 ± 9.7 μg/mL ConA) with 180 uL 2% RBC. ConA/RBC ratio in condition 1 was similar to condition 4 of free ConA assay, while condition 2 was intermediate between conditions 2 and 3 of free ConA assay. Blank IL was used for the negative control. Agglutination with ConA grafted IL was obtained only with condition 2, as shown in Figure 4.

**Figure 5:**
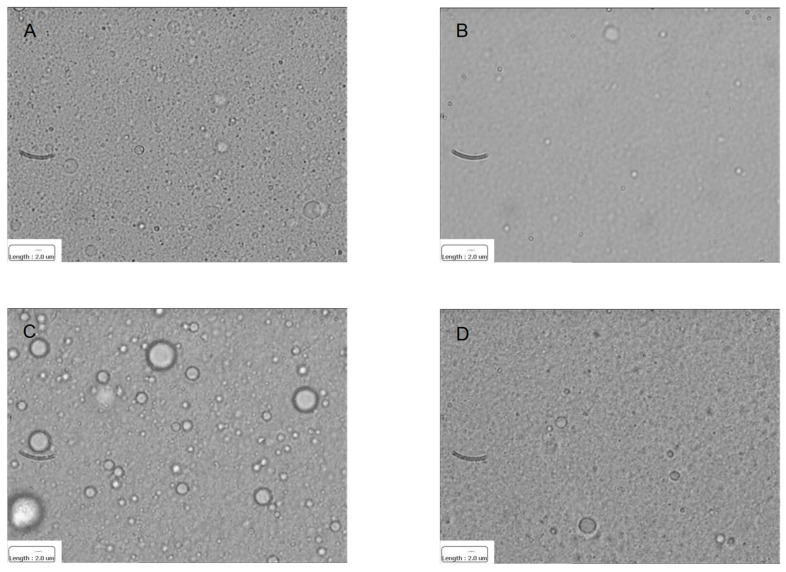
Nanoemulsion coalescence in the presence of 2% starch mixture by optical microscopy. (**A**): ConA grafted IL; (**B**): IL. (**C**): ConA grafted IL with 2% starch mixture: (**D**): IL with 2% starch mixture. Reported scale bar: 2 μm. Abbreviations: ConA: Concanavalin A; IL: 20% Lipofundin^®^ MCT.

The emulsion coalescence after the addition of 2% starch mixture was assessed by DLS (Table 2) and optical microscopy (Figure 5). While blank IL was unaffected by the addition of starch, relevant increase in droplet size was observed for ConA grafted IL. However, given the 5 μm upper size cut-off of DLS, droplet size increase can only be appreciated by optical microscopy.

### 3.3. Permeation Studies through Bovine Nasal Mucosa

**Figure 6:**
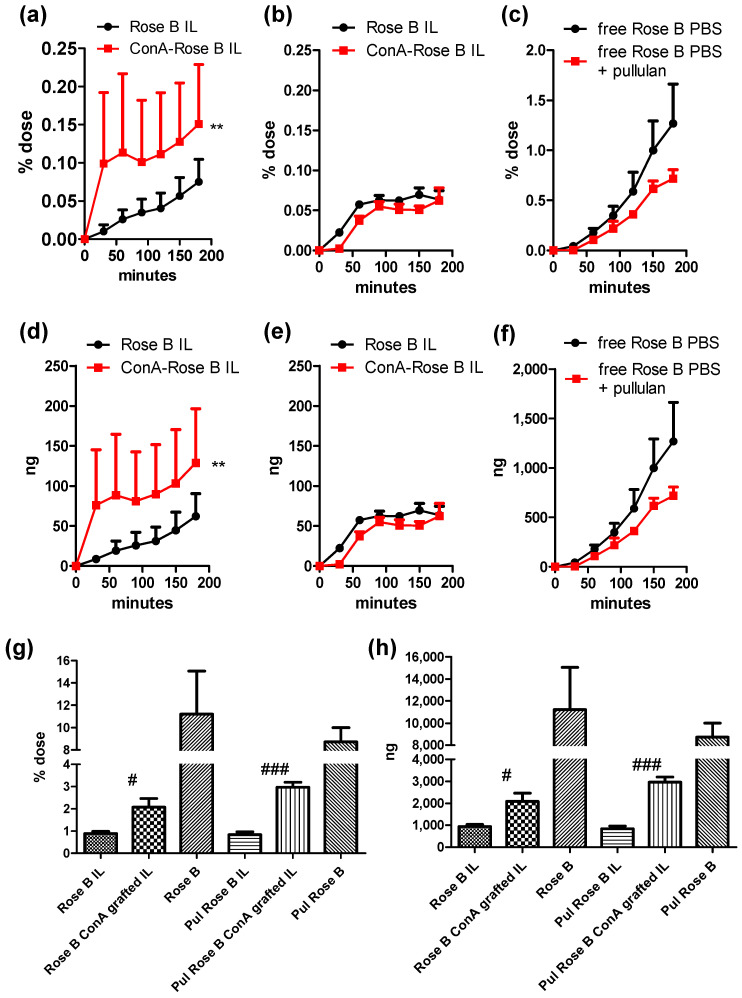
Permeation of Rose Bengal (Rose B) as labelled nanoemulsions (**a**,**b**,**d**,**e**) or as a free drug (**c**,**f**) through bovine nasal mucosa and its accumulation (**g**,**h**). As the receiving phase it was used either PBS (**a**,**d**) or 1% Pullulan (Pul) (**b**,**e**); in (**c**,**f**) panels PBS and 1% Pullulan are compared. Statistical analysis: ** *p* < 0.01 Permeation: ConA grafted Rose B IL vs. Rose B IL. # < 0.05 Accumulation: ConA grafted Rose B IL vs. Rose B IL. ### < 0.0005 Accumulation: Pul ConA grafted Rose B IL vs. Pul Rose B IL.

Results from ex vivo studies, performed on freshly excised bovine nasal mucosa, are shown in Figure 6.

Rose Bengal was selected as the fluorescent probe to mimic the behaviour of potential drug candidates because it is water soluble, yet lipophilic, allowing the loading into the oil matrix. Moreover, owing to its high MW (973 Da), it shows poor permeation through skin and mucosas [25]. However, permeation and accumulation in the nasal mucosa were much greater in the case of the free probe compared to the IL loaded one. This confirms that the fluorescent probe is associated to the lipid matrix, which reduces its availability for permeation and accumulation, meaning that it is suitable to evaluate the enhancing effect on permeation of ConA grafting onto IL.

Moreover, pullulan, used as the chemo-attractant agent, seems to negatively affect the permeation of the free probe, while accumulation is almost unaffected. Therefore, in the case of Rose Bengal labelled nanoemulsions, pullulan chemo-attractant effect can only be appreciated in the accumulation, but not in the permeation. Nonetheless, in the absence of pullulan, ConA grafted IL enhances the permeation of the fluorescent probe compared to unfunctionalized IL, despite the effect of ConA functionalization is highly variable among mucosa specimen, likely due to differences in the glycocalyx composition. Rose Bengal accumulation from nanoemulsions, instead, is enhanced by ConA grafting to a larger extent in the presence of the chemo-attractant agent (almost 2-fold higher Rose Bengal accumulation compared to naïve IL, in the case of PBS and 3-fold in the case of pullulan).

No turbidity from nanoemulsions was detected in the receiving phase, allowing us to hypothesise that intact IL should first be internalized in the mucosal cells, before Rose Bengal release can occur intracellularly, in order to achieve permeation to the receiving phase. The evidence that the permeation, but not the accumulation, was affected by the pullulan addition to the receiving phase could indicate that the chemo-attractant might lead to a disturbance in the cell membrane and cytoplasm (likewise due to an osmotic effect), which would then result in an altered intracellular release and a reduced permeation of the released fluorescent probe. On the other side, Rose Bengal accumulation, which is affected by the chemo-attractant, should be mainly attributed to the cell internalization of the nanoemulsion at the luminal side of the mucosa. Here the presence of the glycocalix allows us to better appreciate the promoting effect of ConA grafting.

## 4. Discussion

Nasal delivery of drugs has several practical advantages for patients, as it is non-invasive, painless, and well-tolerated, allowing multiple administrations, making it suitable for treating chronic diseases. Moreover, a quick onset of action may be obtained, avoiding gastrointestinal and hepatic pre-systemic metabolism. However, as previously mentioned, drug uptake is mainly limited by the short residence time (15–30 min) due to the mucociliary clearance, the low drug permeability, and the small volume available for administration [5,6]. In particular, the effects of formulation engineering on local permeability through the nasal mucosa can be preliminarily evaluated by suitable ex vivo freshly excised nasal mucosa models [26]. To this aim, a suitable device should be employed, capable of maximizing the receiving phase-to-contact area ratio in order to appreciate minimal permeation. This can be obtained by the multi-compartment rotating cell (Figure S1), which also allows the comparison of multiple experimental conditions within the same experiment.

Despite the claimed advantages of lipid nanocarriers to overcome concerns associated with nasal delivery, quick uptake by the paracellular route is probably hampered by their relatively large size [15] and might be improved by exploiting surface functionalization with ligands, capable of binding the mucosal glycocalix, such as ConA [10]. Indeed, ConA can bind to several different types of human airway epithelial cells, and endocytosis of ConA occurs within 1 h at 37 °C [27]. However, despite several articles reporting the enhanced cellular uptake of lectin-conjugated nanoparticles [16,28,29,30], no attempt to formulate ConA grafted injectable nanoemulsions has yet been carried out.

The decoration of lipid nanoparticles with proteins can be performed through different strategies, either conjugating the protein to the lipid first, and then obtaining the nanoparticles, or grafting the protein on the preformed nanocarriers [31]. In order to decorate the precast IL nanoparticles, maleimide–thiol chemistry was employed for ConA grafting for its chemical selectivity, owing to a previously established method. Oxidation of disulfide of thiolated ConA was avoided by working in a mild reducing environment. Furthermore, since an excess of maleimide linker was used with respect to ConA, final saturation was performed with cysteine [17,18]. A noteworthy point: such chemical grafting is associated with a low yield, being an interface reaction. Nonetheless, the grafted ConA is exposed on the IL surface and available for binding with the cell surface. Indeed, ConA grafted IL was capable of RBC agglutination and enhancing permeation and accumulation of IL-loaded Rose Bengal into bovine nasal mucosa in ex vivo experiments.

Lipophilic drugs easily pass through bio-membranes, but they are usually poorly water soluble, and thus need a vehicle to be delivered. In contrast, hydrophilic drugs, especially those with high MW, may not have the ability to cross bio-membranes, meaning that only a small fraction of the administered dose is actually delivered with conventional nasal solutions [15,32]. Rose Bengal is a lipophilic fluorescent probe, as it easily partitions within the inner phase of IL with a high EE%. Nonetheless, it is fairly water soluble, but its relatively high MW prevents it easily permeating biological barriers. Thus, it can be assumed as a model compound capable of recapitulating the main nasal permeation issues of potential drug candidates. Our preliminary findings indicate that nanoemulsions reduce the permeation of the loaded compound, likely due to a oil partition effect. Nonetheless, ConA grafting is capable of increasing both the permeation and accumulation of the loaded compound in the nasal mucosa by means of interaction with the glycocalyx on the luminal side.

Of note, the extent of permeation is much lower than that of accumulation, likely due to the fact that entire droplets might be internalized by cells, followed by intracellular release and translocation of the released compound. However, several processes cannot be fully appreciated in this isolated mucosa model and should be the subject of further in vivo studies. Firstly, ConA is often used as a mitogen to induce in vitro proliferation of lymphocytes (which contain several glycosyl groups), allowing their activation in the nasal cavity and enhancement of the lymphatic transport of nanodroplets in vivo [16]. Moreover, in vivo the mucociliary clearance should affect the residence time of the nanoemulsion to a large extent, likely enhancing the effect of ConA-mediated anchorage of functionalized IL to the nasal mucosa.

## 5. Conclusions

Injectable nanoemulsions are attracting rising interest as lipophilic drug delivery systems due to their excellent biocompatibility. Indeed, this lipid-based formulation can be decorated on the surface with different ligands (proteins, antibodies, peptides etc.) able to bestow specific biological and pharmacological properties (i.e., active targeting, permeation enhancers, etc.). This work is a proof of concept that Concanavalin A-decorated nanodroplets have the potential to be exploited for the intranasal delivery of active molecules.

## Figures and Tables

**Table 1:** Physico-chemical characterization of nanoemulsions. Abbreviations: ConA: Concanavalin A; DLS: dynamic light scattering; EE%: % entrapment efficiency; IL: 20% Lipofundin^®^ MCT; PDI: polydispersity index.

	Rose Bengal		DLS		Z Potential	ConA	
	μg/mL	EE%	Size (nm)	PDI	mV	Grafted μg/mL	% Unreacted
Blank IL	-	-	233.2	0.110	−35.07 ± 3.89	-	-
	100	74.5 ± 4.1	230.2	0.139	−38.74 ± 1.35	-	-
ConA grafted IL	-	-	231.7	0.058	−36.47 ± 2.04	64.6 ± 9.7	43.9 ± 2.8
	100	82.9 ± 3.6	250.3	0.114	−37.55 ± 6.36	64.6 ± 9.7	43.9 ± 2.8

**Table 2:** Nanoemulsion coalescence in the presence of 2% starch mixture by DLS. Abbreviations: ConA: Concanavalin A; DLS: dynamic light scattering; IL: 20% Lipofundin^®^ MCT; PDI: polydispersity index.

	ConA Grafted IL		IL	
2% Starch Mixture Volume (μL)	Mean Size (nm)	PDI	Mean Size (nm)	PDI
0	249.5	0.066	217.8	0.088
60	257.8	0.103	226.0	0.103
120	255.2	0.158	227.9	0.026

## Data Availability

The raw data supporting the conclusions of this article will be made available by the authors on request.

## Supplementary Materials

The following supporting information can be downloaded at: https://www.mdpi.com/article/10.3390/ma17204959/s1, Figure S1. Photographs of the Multi-compartment rotating cell.
