# Supplementary material for: Concanavalin a Grafted Nanoemulsions for Nasal Delivery: Preliminary Studies with Fluorescently Labelled Formulations
Source: Materials (Basel). 2024 Oct 11;17(20):4959. doi: 10.3390/ma17204959 (PMC11509158; doi:10.3390/ma17204959)
Supplement: Supplementary file 1 [file materials-17-04959-s001.zip › materials-3190737-supplementary.pdf]

Concanavalin A Grafted Nanoemulsions for Nasal Delivery: Preliminary Studies with Fluorescently Labelled Formulations

Merve Mışraklı, Sebastiano Antonio Rizzo, Valentina Bordano, Annalisa Bozza, Luca Ferraris, Elisabetta Marini, Elisabetta Muntoni, Maria Teresa Capucchio, Anna Scomparin and Luigi Battaglia

Supplementary Materials

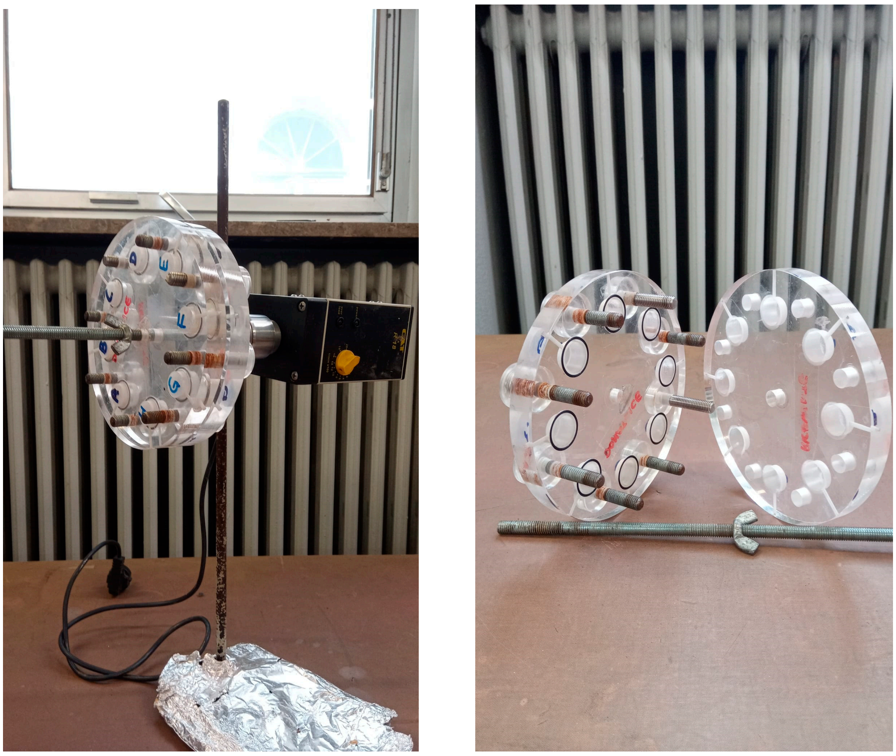

Figure S1. Photographs of the Multi-compartment rotating cell
